# Self-Assembled Hydrogel Based on (Bio)polyelectrolyte Complex of Chitosan–Gelatin: Effect of Composition on Physicochemical Properties

**DOI:** 10.3390/gels10120786

**Published:** 2024-12-01

**Authors:** Kashurin Aleksandr, Litvinov Mikhail, Podshivalov Aleksandr

**Affiliations:** Center for Chemical Engineering, ITMO University, Kronverkskiy Prospekt, 49, 197101 Saint-Petersburg, Russia; pkashurin@mail.ru (K.A.); mikhail.litvinov.1996@mail.ru (L.M.)

**Keywords:** chitosan, gelatin, polyelectrolyte complexes, hydrogel, dynamic mechanical analysis, sorption analysis

## Abstract

Taking into account the trends in the field of green chemistry and the desire to use natural materials in biomedical applications, (bio)polyelectrolyte complexes ((bio)PECs) based on a mixture of chitosan and gelatin seem to be relevant systems. Using the approach of self-assembly from the dispersion of the coacervate phase of a (bio)PEC at different ratios of ionized functional groups of chitosan and gelatin (*z*), hydrogels with increased resistance to mechanical deformations and resorption in liquid media were obtained in this work in comparison to a hydrogel from gelatin. It was found that at *z* ≥ 1 a four-fold increase in the elastic modulus of the hydrogel occurred in comparison to a hydrogel based on gelatin. It was shown that hydrogels at *z* ≈ 1 had an increased sorption capacity and water sorption rate, as well as increased resistance to the in vitro model environment of phosphate-buffered saline (*PBS*) solution containing lysozyme at 37 °C. It was also shown that in *PBS* and simulated gastric fluid (*SGF*) solutions, the effect of the polyelectrolyte swelling of the hydrogels was significantly suppressed; however, at *z* ≥ 1, the (bio)PEC hydrogels had increased stability compared to the samples at *z* < 1 and based on gelatin.

## 1. Introduction

To date, tissue engineering continues to develop in the direction of designing new materials that meet the requirements for safe and effective use in medicine. A significant number of studies focus on creating biocompatible chemically cross-linked hydrogels. This class of materials is one of the most traditional and extensively studied, although many works are still devoted to studying the properties of already-known systems modified with new fillers [1,2,3], including various types of cross-linking agents [4,5,6], among others. An alternative approach involves creating hydrogels where the cross-linking of polymer chains is based on physical interactions, particularly electrostatic interactions [7,8,9]. The primary strategy for producing such materials is to obtain a mixture of individual polyelectrolyte solutions. By varying the pH of the medium, it becomes possible to achieve the ionization of oppositely charged functional groups, leading to their subsequent self-assembly into complex supramolecular structures based on polyelectrolyte complexes (PECs). The ability to control the structure and morphology of these materials explains researchers’ interest in developing PEC-based materials for various medical applications, including implants for volume enhancement and soft tissue regeneration [10,11], scaffolds that stimulate cell growth [12,13], and containers for targeted drug delivery [14,15]. Additionally, the advantages of these materials over chemically cross-linked hydrogels are due to the simplified technology of their production, which reduces the number of synthesis stages and eliminates the need to use toxic organic solvents.

An important feature of PEC-based hydrogel materials that classifies them as “smart” systems is their ability to respond specifically to a wide range of stimuli from the external environment. This feature is regulated during the composition and technological aspects of synthesizing hydrogel, allowing for the optimization of their sensitivity to desired physical and/or chemical actions. For example, in the work [16], the formation of a pH-sensitive PEC gel based on chitosan and sodium alginate was demonstrated. It has been shown that the mechanical characteristics of such gels depend on the molar ratio of polymers. Moreover, the maximum elastic modulus of the gel was 7 kPa, achieved at an equimolar ratio of ionized groups. According to the authors, this was associated with the highest degree of electrostatic interaction, leading to the increased entanglement of cross-linked polymer chains. Another study, [17], evaluated the properties of gels based on chitosan and carrageenan obtained through electrostatic interaction over a wide pH range and at an equimolar ratio of the polymers. It was shown that the greatest electrostatic interaction for this PEC composition occurred at pH 4–5. Such synthesis conditions led to the formation of strong internal cross-linking within the gels, which was reflected in an increase in the product yield, viscosity, and fiber content that formed the basis of the hydrogel material.

An illustrative example of the design of hydrogel composition properties is found in the work [18], which studied the changes in the properties of polymer systems based on the sodium salt of N-succinyl chitosan (NSC) and poly-N,N-diallyl-N,N-dimethylammonium chloride (PDADMAC) depending on the composition of the reaction mixture and the conditions of PEC preparation. It was found that at an equimolar ratio of oppositely charged functional groups (*z* = 1), a denser intermolecular network with fewer defects and hydrophilic groups that did not participate in cooperative interactions were formed. This effect directly influenced the behavior of the hydrogel material in sorption and mechanical analysis. Specifically, increasing the molar ratio from *z* = 0.5 to *z* = 1 resulted in a two-fold decrease in the swelling rate constant, while the critical strain and storage modulus in the amplitude test increased significantly.

As mentioned above, the structural component of polyelectrolyte complexes (PECs) is one of the key parameters responsible for the materials’ characteristics. This explains the recent increased interest among researchers in PECs consisting of polyampholyte-polycation/polyanion combinations [16,19]. In such combinations of polyelectrolytes, the potential for controlling the structure and, consequently, the properties of the material are fully realized. This characteristic of the mentioned PEC combinations is evident due to the wide range of ratios of polyelectrolytes involved in complex coacervation, allowing for a more precise regulation of the total charge on the surface of the resulting material. From this perspective, two of the most attractive polymers for creating PECs are chitosan and gelatin. In these PECs, electrostatic interactions occur between the positively charged amino groups in the glucosamine links of chitosan and the negatively charged carboxyl groups in the glutamine and asparagine amino acid residues of gelatin with the accompanied release of counterions, leading to an increase in the entropy of the system [20]. During this interaction, intermediate supramolecular aggregates are initially formed, which subsequently regroup into coacervate particles. When approaching the stoichiometric charge balance in the system, there is a decrease in the electrostatic repulsion between ionized groups of the same charge. This, in turn, favors the self-assembly of complex supramolecular structures due to other non-electrostatic interactions. As a result of these interactions, a separation into colloid-rich and colloid-poor phases is observed.

The formation of PEC structures between chitosan and gelatin in solution is limited by the isoelectric point (IEP, *pI*) of gelatin and the protonation constant (*pK*_0_) of chitosan. As a result, electrostatic interactions leading to the formation of stable PEC structures (coacervate dispersions) are possible under the following condition: *pI* < pH < *pK*_0_ [21]. Another challenge in obtaining stable complexes in this system occurs due to the low content of glutamic and aspartic acids in gelatin. These amino acids can dissociate to form negatively charged functional groups. As noted in [22], in the case of gelatin obtained from bovine skin, there are approximately 51 amino acid residues capable of carrying a negative charge per 1000 amino acid residues. In contrast, gelatin from pig skin contains about 124 such residues. This characteristic of gelatin’s amino acid composition necessitates using it in excess within the chitosan–gelatin system when forming PECs to balance the number of oppositely charged functional groups, as also mentioned in [23].

All this leads to the conclusion that there are few studies in the modern scientific literature focused on the production of hydrogels based on polyelectrolyte complexes (PECs) between chitosan and gelatin, as well as on the investigation of their structural properties. For example, in the work [24], it was found that under low-concentration conditions, PEC structures based on chitosan and gelatin exhibited a correlation between the rheological properties of the obtained hydrogels and the concentration of the chitosan solution. Increasing the concentration of chitosan in the range of 0.1–0.6% (with a constant gelatin concentration of 1.0%) resulted in an exponential increase in both the yield strength and apparent viscosity of the hydrogel material. The authors attributed this phenomenon to the strengthening of the gels due to the formation of new nodes in the structural network, which arose from electrostatic and hydrogen interactions between the biopolymers. However, in the study, the hydrogel production was conducted at pH < *pI*, where the formation of stable dispersions of (bio)PEC coacervates was a difficult task. In another study, [25], it was demonstrated that the formation of coacervate dispersions in a mixture of chitosan and type B gelatin solutions at a 1:2 ratio was accompanied by a significant increase in solution turbidity, with maximum values observed at pH = 5.5. Additionally, during the prolonged sedimentation of the coacervate dispersion, phase separation occurred, resulting in a precipitate that was a thermally reversible colloidal gel characterized by a sol–gel transition at a temperature of 42 °C.

The thermoreversible properties of hydrogels were also considered in the work [26], which proposed the use of chitosan–gelatin hydrogels for skin bioprinting. The authors of the study demonstrated a pH-dependent crosslinking approach that was used to create a material capable of retaining a gel-like state in the temperature range of 20–40 °C. It was also found that the viscosity of hydrogels containing gelatin solutions with concentrations of 2.5%, 5.0%, and 7.5% increased by 9, 22, and 30 times, respectively, at shear rates ranging from 0.1 to 100 s^−1^.

An analysis of the scientific literature shows that most of the work in this field is devoted to the production of (bio)PEC hydrogels from chitosan and gelatin solutions at their sol–gel transition. In the presented works, the influence of pH values and polymer concentrations on the self-assembly and rheological properties of (bio)PEC hydrogels are studied. At the same time, little attention is paid to the study of the process and conditions of the self-assembly of (bio)PEC hydrogels in mixtures of chitosan and gelatin solutions. The influence of the ratios of polyelectrolytes and their ionized functional groups on the conditions of self-assembly, as well as the viscoelastic and sorption properties of hydrogel materials, has also been insufficiently studied. In our previous work [27], the effect of the polyelectrolyte ratio on the structure of (bio)PEC coacervate dispersions (hydrogel precursors) in mixtures of dilute chitosan and gelatin solutions was partially investigated. Using optical microscopy and dynamic light scattering methods, it was shown that with an increase in the ratio of polyelectrolytes, the efficiency of the polyelectrolyte complexation process increased, which was accompanied by a compactization of the (bio)PEC particle sizes. However, the transition from (bio)PEC dispersion in dilute solutions to self-assembling hydrogels with an increased concentration of polymers remains unclear. Moreover, the dependence of the self-assembly process of hydrogels, as well as their viscoelastic behavior, sorption properties, and resistance to liquid media, on the ratio of the ionized functional groups of polyelectrolytes also remains unknown.

In this regard, the purpose of this work was to study the influence of the ratio of ionized functional groups of chitosan and gelatin in solution on the process of the self-assembling of (bio)PEC hydrogel structures and their physicochemical properties. In the work, the obtained (bio)PEC hydrogels were studied by the methods of attenuated total reflectance Fourier-transformed infrared (ATR-FTIR) spectroscopy, scanning electron microscopy (SEM), and dynamic mechanical analysis (DMA) of viscoelastic behavior. Much attention was paid to the study of the process and kinetics of the sorption of water and model liquid buffer solutions, as well as in vitro resorption in a phosphate-buffered saline solution with the addition of lysozyme at a human body temperature (37 °C).

## 2. Results and Discussion

### 2.1. Self-Assembly of Chitosan–Gelatin (Bio)PEC Hydrogels

**Figure 1:**
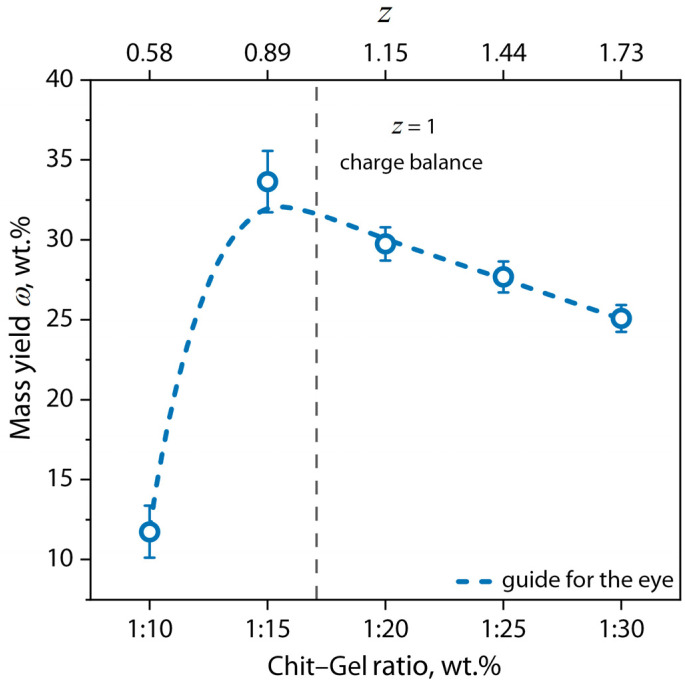
Mass yield of Chit–Gel (bio)PEC hydrogels versus *z* value.

The dependence of the mass yield of the (bio)PEC hydrogels on the *z* value is shown in Figure 1.

Figure 1 shows that the mass yield of the obtained (bio)PEC hydrogels depended on the ratio of biopolymers and values of *z*. When the ratio of Chit–Gel was 1:10 (*z* = 0.58), there was a minimum yield of hydrogel. This effect may be due to the predominance of a positive charge on the surface of (bio)PEC particles at this *z* value because non-stoichiometric interaction between chitosan and gelatin partially complicates the process of the self-assembly of the hydrogel. With an increase in the proportion of negatively charged carboxyl groups in the solution to *z* ≈ 1, the yield of the hydrogel tended to a maximum, which indicated an increase in the efficiency of polyelectrolyte binding between the polymer macromolecules. These effects are described in detail in our previous work [27]. As we assumed, this phenomenon can be associated with an increase in the isotropy of the process of electrostatic interaction between oppositely charged ionized groups, which in turn leads to a decrease in the concentration of polymers in the equilibrium phase. This is due to stabilizing by the effects of electrostatic repulsion from homonymous charges.

**Figure 2:**
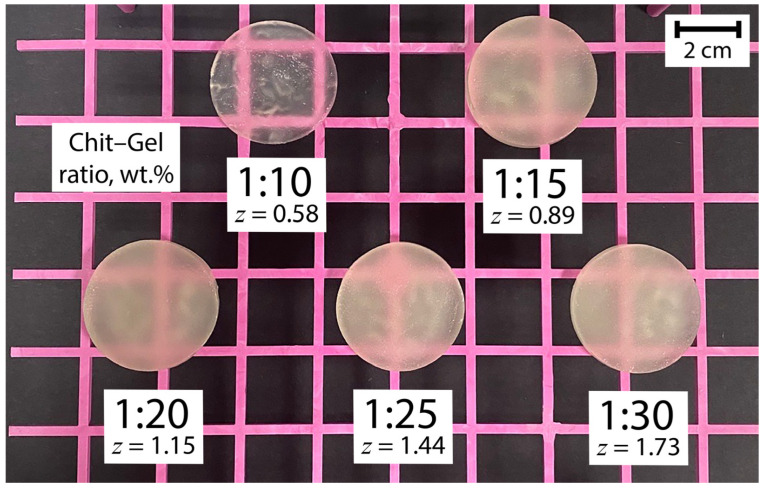
Appearance of self-assembled Chit–Gel (bio)PEC hydrogels at shown *z* values.

Further, in order to form semi-solid structures of (bio)PEC hydrogels, it is necessary to remove the temperature influence (35 °C) exerted on the system until typical phase separation appears with the formation of equilibrium (colloid-depleted) and coacervate (colloid-enriched) phases [28], and it is also necessary to store the solution at room temperature (25 °C) until the gelatinization process is completed. Figure 2 shows the appearance of (bio)PEC hydrogels at different values of *z*.

It is noteworthy that the appearance of the hydrogels did not differ much when *z* was varied, except for the sample with *z* = 0.58. This sample looked more transparent compared to the others, and we believe that this effect was observed due to the low concentration of (bio)PEC dispersion in the solution during hydrogel self-assembly, which led to a low concentration of scattering centers in the material. This effect may also have been partly due to some difference in the thickness of the sample due to the minimum mass yield of the product (Figure 1). When the *z* value increased, with the increase in the mass yield of the semi-solid phase of the hydrogel, significant visual scattering was observed in the obtained hydrogel samples. This observation also indicated an increase in the number of scattering centers in the structure of materials representing the particles of the coacervate phase of the (bio)PEC.

### 2.2. Morphology of Chitosan–Gelatin (Bio)PEC Hydrogel Lyophilizates

**Figure 3:**
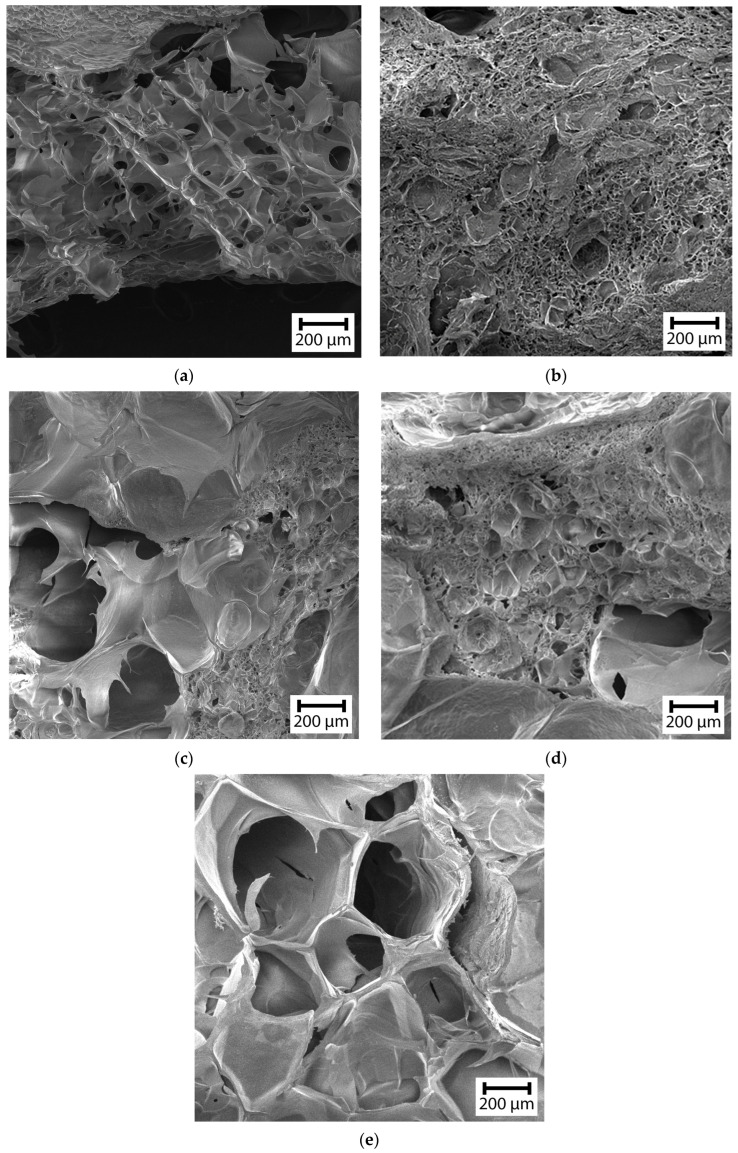
SEM microphotographs of Chit–Gel (bio)PEC hydrogels at (**a**) *z* = 0.58, (**b**) 0.89, (**c**) 1.15, (**d**) 1.44, and (**e**) 1.73.

The surface morphology of the obtained lyophilized (bio)PEC hydrogels is shown in electron microphotographs in Figure 3.

Visual analysis of the morphology of the lyophilizates showed a highly porous structure typical for hydrogels [29,30,31]. The presence of interconnected pores distributed over the entire volume of the hydrogels obtained was observed. In general, it was evident that for all Chit–Gel ratios, large-diameter pores of more than 200 μm in diameter were observed in the hydrogel structure. However, the sizes of these pores were practically independent of the Chit–Gel ratio, except for the mixture at *z* = 1.73. Thus, the pore diameter for the mixture lay in the range of 51–138 μm at *z* = 0.58; of 56–151 μm at *z* = 0.89; of 43–259 μm at *z* = 1.15; of 54–157 μm at *z* = 1.44; and of 153–463 μm at *z* = 1.73, respectively. Despite this, for compositions around *z* ≈ 1 (Figure 3b–d), a dense network of a large number of pores with a diameter several times smaller was also observed. In these cases, the small pores had, on average, diameters of 17.9 + 4.7 μm at *z* = 0.89; of 14.8 + 2.9 μm at *z* = 1.15; and of 12.7 + 3.6 μm at *z* = 1.44, respectively. Probably, such morphology was due to the process of balancing the surface charge of (bio)PEC coacervates during the self-assembly of the hydrogel structure. Despite this, for the hydrogel at *z* = 1.73 (Figure 3e), a structure with only large-diameter pores was characteristic. As will be shown below, such differences in the morphology of the hydrogels did not have a significant negative effect on the viscoelastic properties and stability of the hydrogels to liquid media. Thus, it can be concluded that the polyelectrolyte interactions of chitosan and gelatin during the self-assembly of hydrogels in solution have a more significant influence on the physicochemical properties of such structures, rather than their spatial structure.

### 2.3. Chemical Composition of Chitosan–Gelatin (Bio)PEC Hydrogels

**Figure 4:**
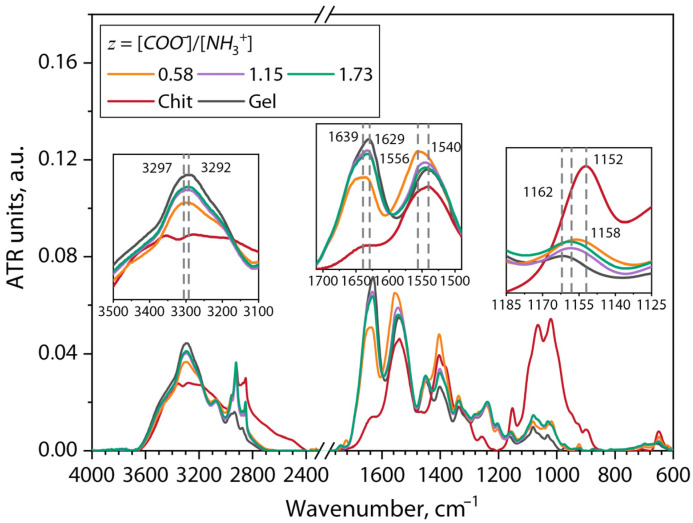
ATR-FTIR spectra of Gelatin, Chitosan, and Chit–Gel (bio)PEC hydrogels at different *z* values.

Figure 4 shows the ATR-FTIR spectra of the Gel, Chit, and Chit–Gel (bio)PEC hydrogels at different *z* values.

Analysis of the chemical composition of the initial biopolymers showed the presence of their typical absorption bands. A detailed description of used chitosan and gelatin is presented in our previous work [27]. In this work, we would like to highlight the most significant absorption bands that can be used to describe the polyelectrolyte interaction between the chitosan and gelatin at different *z* values. In the spectra of both polymers, a broad absorption band in the range of 3600–2700 cm^−1^ associated with the valence vibrations of -OH and -NH groups was observed [32]. Also, characteristic absorption peaks of amide-I and amide-II with centers at 1629 and 1540 cm^−1^, respectively, were observed for the gelatin sample [33]. As for chitosan, the location of the amide-I and amide-II absorption bands were at the wavenumbers of 1635 and 1540 cm^−1^, respectively [32,34]. The band at 1162 cm^−1^ was due to the presence of carboxyl groups in glutamic and aspartic acid residues in the gelatin macromolecule [35]. Finally, the band at 1152 cm^−1^ was due to asymmetric C-O-C valence vibrations, which are characteristic of the saccharide structure of chitosan [36].

Under the analyzed conditions at pH = 6, oppositely charged ionized groups of chitosan and gelatin preferentially interacted by an electrostatic mechanism, which made a significant contribution to the structural features of the polyelectrolyte complex. As can be seen from the presented spectra, a shift in the position of the amide-A peak centered at 3292 cm^−1^ for the gelatin towards higher wavenumbers for the Chit–Gel samples centered at 3297 cm^−1^ was observed. This hypochromic shift effect has also been observed in another works [33,37] and has been attributed to a reduction in bond length for the –NH group because of electrostatic interaction between the polymers. In addition, a decrease in the intensity of this broad absorption band was observed for the Chit–Gel hydrogel compared to the gelatin, which was attributed to a decrease in the interaction of –NH and –OH with H_2_O as a result of conformational rearrangements in the macromolecules [32].

Also indicative of the character of the interaction between the polymers were the absorption bands of amide-I and amide-II with centers at 1629 and 1540 cm^−1^. It was noted that for all presented spectra of the Chit–Gel samples, the intensity of the amide-I peak decreased and there was a shift in the amide-II peak to the short-wave region (from 1540 to 1556 cm^−1^) compared to the spectrum for the native gelatin. This effect has been observed in a number of works [38,39,40], which is attributed to a decrease in the number of disordered structures in macromolecular chains of gelatin as a result of the electrostatic binding reaction of ionized groups.

The main countershift was observed for a wide chitosan peak with a center of 1152 cm^−1^ and a band of 1162 cm^−1^, corresponding to valence fluctuations of carboxyl groups in the composition of the gelatin amino acids to the intermediate frequency of the spectrum of the Chit–Gel samples centered at 1158 cm^−1^. The results obtained show that the electrostatic interaction of ionized, oppositely charged macromolecules of the polymers with the formation of (bio)PEC structures occurred under selected conditions. Similar results were obtained in works [32,35], which also investigated the formation of polyelectrolyte complexes between chitosan and gelatin.

### 2.4. Viscoelastic Properties of Chitosan–Gelatin (Bio)PEC Hydrogels

**Figure 5:**
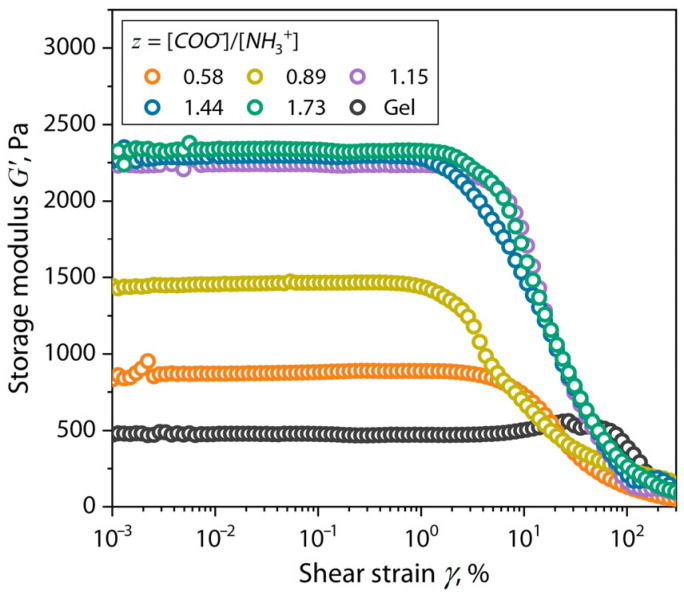
*G*′ versus *γ* profiles for Gel and Chit–Gel (bio)PEC hydrogels at different *z* values at temperature of 25.0 ± 0.2 °C.

Figure 5 shows the profiles of *G*′ for the (bio)PEC hydrogels at different *z* values, as well as the Gel hydrogel.

Figure 5 shows the typical curves of the storage modulus for the gel-like structures. For all the compositions studied, a region of a linear viscoelasticity range (LVE range) can be distinguished, where the modulus demonstrates a constant value. After having reached the limit of linear viscoelasticity (LVE limit) at increased values of shear strain, the viscoelastic structure of the sample is destroyed and the values of *G*′ decrease. For the studied compositions, we observe a clear relationship between the values of the *G*′ in the LVE range, LVE limit values, and *z* values of the polymers in the solution after hydrogel preparation. Therefore, based on the DMA data, according to Equations (2) and (3), the distance between the crosslinking points, ζ, and the crosslinking density, *n_e_*, in the gel-like material were calculated. The values of these parameters are shown in Table 1.

The values of the moisture content of the (bio)PEC hydrogel samples presented in the table, obtained by thermogravimetric analysis of moisture evaporation at the first stage of the mass loss of the samples, show a deviation of within 4%. Such a difference in the moisture content of the samples is insignificant and allows us to exclude its influence on the structure and physical and mechanical properties of the samples studied. It can be seen from the table that with an increase in the proportion of the gelatin and, correspondingly, the *z* value of the hydrogel, there was an increase in the *G_e_*′ value in the LVE-range. As the *z* value increased to 1.15, an increase in the value of *G_e_*′ and the crosslinking density was observed by about three times, and the distance between the crosslinking points decreased by 43% in the (bio)PEC hydrogels. However, increasing the value of *z* above 1.15 (or *z* > 1) did not lead to significant changes in the above parameters. This behavior showed that with an increase in the value of *z* to 1, a significant increase in the density of the polymer network occurred, which led to an increase in the elasticity of the entire structure of the material due to the balancing of the charge on the surface of the (bio)PEC coacervates in the solution. At the same time, when the charge balance reached (*z* = 1) and the ratio of *z* > 1 increased further, effective compaction of the polymer network did not occur. In addition, for each of the compositions of the (bio)PEC hydrogels, an increased value of *G_e_*′ relative to the Gel hydrogel was observed, which further emphasized the role of polyelectrolyte interaction in the formation of the elastic structures. In addition, as mentioned above, the highly porous structure of the hydrogels at *z* > 1 (Figure 3) did not have a negative effect on their viscoelastic behavior, which was associated with a decrease in ζ and an increase in *n_e_* values.

### 2.5. Swelling Behavior of Chitosan–Gelatin (Bio)PEC Hydrogels

Sorption analysis makes it possible to characterize the structure and properties of a hydrogel material with the modeling of swelling conditions, which is reflected in changes in the degree of swelling and liquid absorption kinetics. By varying the solvent when analyzing the sorption capacity of hydrogel samples, different models of in vitro liquid uptake are considered.

**Figure 6:**
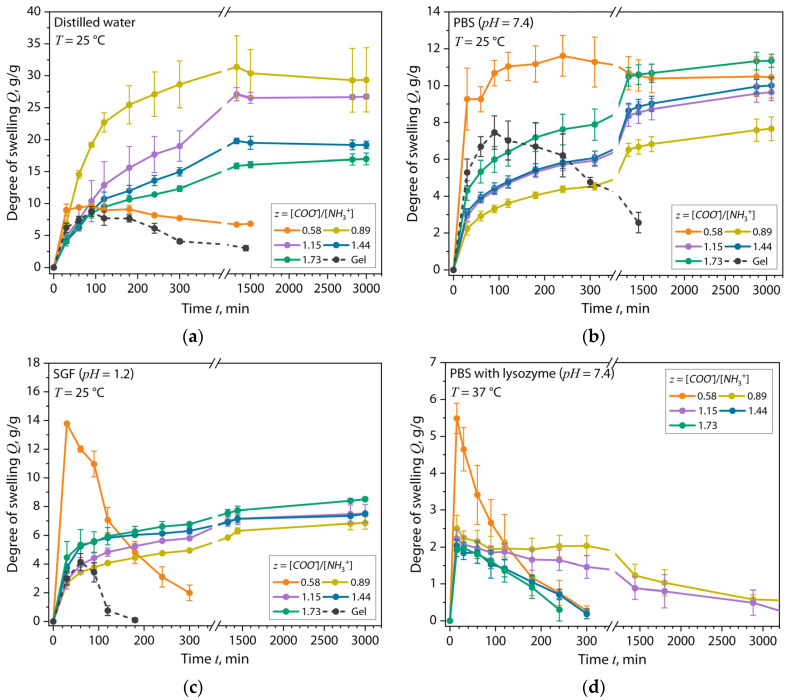
Sorption curves of Gelatin and Chit–Gel (bio)PEC hydrogel lyophilizates in (**a**) distilled water (pH = 6) at 25 °C, (**b**) *PBS* solution (pH = 7.4) at 25 °C, (**c**) *SGF* solution (pH = 1.2) at 25 °C, and (**d**) *PBS* with lysozyme solution (pH = 7.4) at 37 °C.

First of all, when analyzing the sorption properties of (bio)PEC hydrogels, it is necessary to take into account many parallel processes that directly affect the kinetics of the sorption process and the sorption capacity of the materials. Firstly, the polyelectrolyte macromolecules themselves can swell or collapse in aqueous solutions depending on the pH and ionic strength of the media. Secondly, in aqueous environments, the formed (bio)PEC structures are held mainly by ionic bonds between the basic and acidic side groups in the polyelectrolyte structure, as well as by weak hydrogen bonds that gradually break down, which leads to a loss of hydrogel mass. For these reasons, the significance of the change in the mass of the (bio)PEC hydrogel during the sorption of an aqueous solution and the kinetics of this process depend on the following factors: (1) the number of free ionized groups on the hydrogel surface; (2) the number of formed ionic bonds between the polyelectrolyte macromolecules (complexation efficiency); (3) the number of hydrogen bonds between the macromolecules; and (4) the ionic strength and pH of the medium. The gravimetric method for estimating the moisture absorption degree of hydrogels was used to study the kinetics of the swelling process of materials depending on the *z* value. Figure 6 shows the kinetic curves of swelling in distilled water, *PBS* and *SGF* solutions, and in a system with *PBS* and lysozyme.

Figure 6a shows that in the sorption analysis of the hydrogels in distilled water, the Gel and Chit–Gel (bio)PEC samples at *z* = 0.58 had sorption capacities of 8.7 g/g and 9.6 g/g, respectively, and reached these values within 90 min of the test, after which a significant loss of mass in these samples occurred. These effects were observed due to the active process of polyelectrolyte swelling, on the one hand, and the low efficiency of complexation in the (bio)PEC sample, on the other hand. In these cases, there were no ionic complex bonds in the Gel sample, while there were very few of them in the (bio)PEC sample at *z* = 0.58, and the structure of the samples was maintained due to hydrogen bonds, which were destroyed during the polyelectrolyte swelling. With an increase in the *z* value for the hydrogels based on the (bio)PEC from 0.58 to 1.73, a significant increase in the sorption capacity of the water occurred, and the loss of sample mass was suppressed throughout the test. The increase in the stability of these samples was due to an increase in the number of ionic complex bonds with an increase in the *z* value, which were stronger than hydrogen bonds and allowed the structure of the samples to be maintained. Despite this, it is clear that with an increase in the *z* value, the sorption capacity of the hydrogels decreased significantly (Table 2). This effect was also a consequence of an increase in the number of ionic bonds between the polyelectrolytes and a decrease in the distances between the nodes of the polymer network in the hydrogel (Table 1). As a result, the degree of freedom for filling the free volume of intermolecular space for water molecules decreased. This feature has also been noted in a number of works [41,42]. Thus, the obtained results of sorption analysis were confirmed by earlier studies of the elastic properties of the (bio)PEC hydrogels, where an increase in the crosslinking density of the material was shown.

Figure 6b shows the results of the sorption test of the hydrogel samples in the *PBS* solution. It is evident that, in general, compared to the test in distilled water, the sorption capacity of the (bio)PEC samples in the buffer solution at *z* ≥ 0.89 decreased by more than two times. This behavior was due to the suppression of the polyelectrolyte swelling of hydrogels due to the charge screening of bound protons in the hydrogel volume. In this case, due to the increased ionic strength of the solution compared to the distilled water, the polyelectrolyte macromolecules “dried out” and collapsed, and the diffusion of the liquid became more complicated. Also, due to this process, the (bio)PEC hydrogel sample at *z* = 0.58 lost its mass less significantly. It is worth noting that in the *PBS* solution, with an increase in the value of *z* ≥ 0.89 in the hydrogel, the sorption capacity of the samples, on the contrary, increased compared to the test in water. Here, it can be said that the sorption capacity depended mainly on the number of charged carbonyls on the surface of the hydrogel, which increased with an increasing *z* value and was reduced due to low-molecular cations in the solution. A similar behavior of polyelectrolyte structures was noted in the work [43], where the authors determined the release of chondroitin sulfate from the PEC composition of chondroitin sulfate/chitosan during the swelling of the hydrogel, including under conditions of the deprotonation of *D*-glucosamine units of chitosan using high-performance liquid chromatography.

In the *SGF* medium (Figure 6c), which is a model medium for human gastric juice, the lowest *Q_max_* value and sample lifetime were observed for the Gel sample, which was associated with a high level of polyelectrolyte swelling at a low pH and the absence of any bonds other than hydrogen bonds that held the structure of the sample. For the Chit–Gel (bio)PEC hydrogels at *z* ≥ 0.89, a decrease in sorption capacity of the hydrogels by more than two times compared to the test in water, which was also slightly lower than in sorption tests in the *PBS* solution, was observed. This effect had the same cause as the effect of the suppression of the polyelectrolyte swelling (collapse) of the hydrogels during tests in the *PBS* solution. It is interesting that in the *SGF* medium the (bio)PEC hydrogel at *z* = 0.58 had the highest *Q_max_* value compared to all the studied hydrogels, regardless of the composition of the liquid medium. At the same time, after reaching this value, an intensive process of the mass loss of this sample began, which led to its complete dissolution after 300 min of the test. Such behavior corresponded to the unlimited swelling (dissolution) of this sample in the *SGF* medium and was associated with a low amount of ionic polyelectrolyte crosslinks of the (bio)PEC, a high value of ζ, and a low value of *n_e_* in the sample. This is because in an *SGF* medium at pH = 1.2 the number of free charged amino groups of gelatin will increase with an increasing *z* value, provided that the pH is below *pI*. For samples with *z* ≥ 0.89, despite the acidic pH of the *SGF* medium, a relatively low loss of hydrogel mass was observed, which also proved the efficiency of the formation of (bio)PEC structures at these polymer ratios.

In vitro studies of the enzymatic resorption of hydrogel samples in a solution of *PBS* with lysozyme at 37 °C (Figure 6d) showed that under these conditions the character of sorption changed relative to other media. It is worth noting that the mass loss of the gelatin samples under these conditions could not be estimated due to their rapid destruction. Two stages of sample mass change could be distinguished: a short (20–40 min) stage of sample swelling and a long stage of sample resorption with the loss of its mass because of structural destruction. In this case, such behavior was caused, on the one hand, by the presence of lysozyme in the medium, which led to the degradation of *β*-glycosidic bonds in the structure of the chitosan, and, on the other hand, by the increased temperature of the dough, approximately equal to the temperature of the human body. It is worth noting that the character of the mass loss of the sample depended on the *z* values. For example, at *z* = 0.89 and 1.15, the stability of the sample increased; the samples were slowly resorbed for 2 days. At the same time, we did not observe a strong loss of mass in these samples in the first 300 min of the experiment. This once again proved the fact that the Chit–Gel (bio)PEC hydrogels at *z* ≈ 1 had the greatest resistance to the aggressive conditions of the liquid medium.

### 2.6. Swelling Kinetics of Chitosan–Gelatin (Bio)PEC Hydrogels

**Figure 7:**
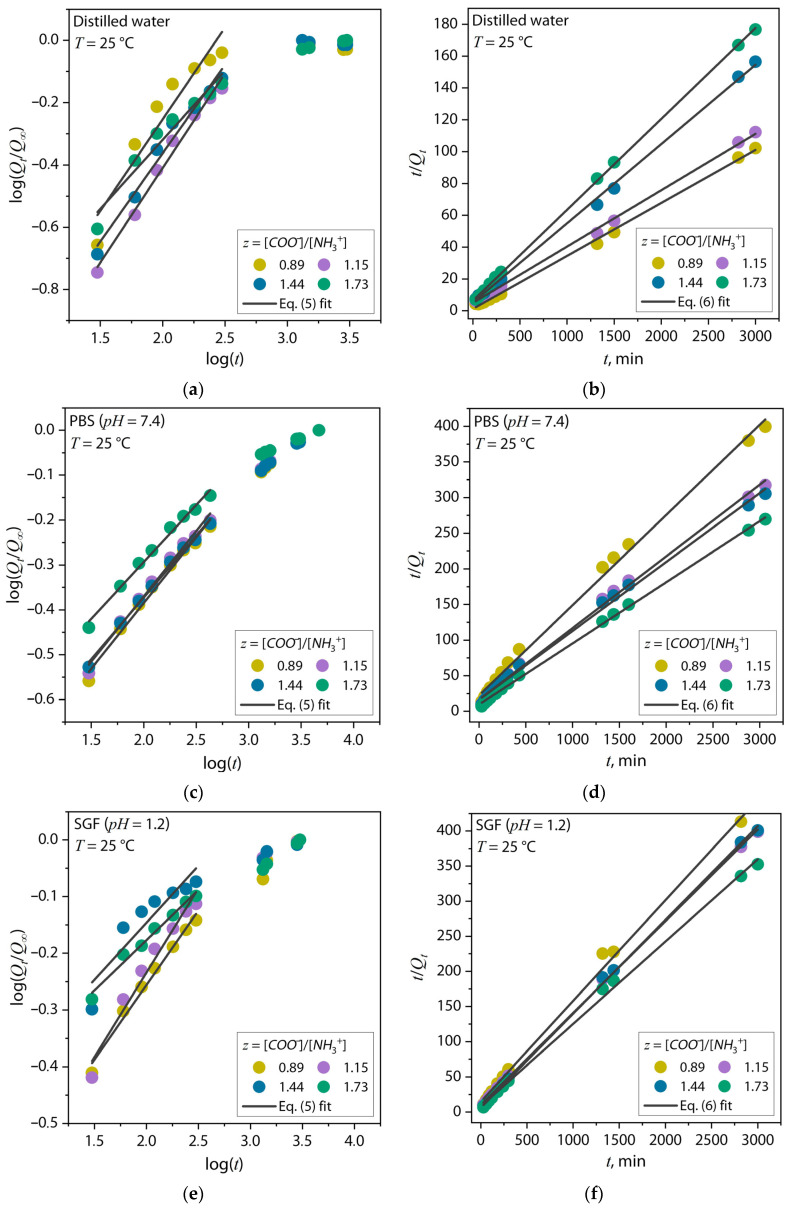
Kinetic dependences of liquid sorption process of Chit–Gel (bio)PEC hydrogel lyophilisates in (**a**,**b**) distilled water and (**c**,**d**) *PBS* and (**e**,**f**) *SGF* solutions and their analytical fitting with Equations (5) and (6), respectively.

To determine the kinetic parameters of the sorption process of the Chit–Gel (bio)PEC hydrogel lyophilizates in water and *PBS* and *SGF* solution, graphs were plotted according to Equations (5) and (6), which are shown in Figure 7. The numerical values of the obtained parameters are shown in Table 2.

As shown in Table 2, in the case of the swelling of the (bio)PEC hydrogels in distilled water, the diffusion coefficient *n* for all (bio)PEC compositions except *z* = 1.73 was in the range of 0.5 > *n* > 1, which corresponded to an anomalous diffusion mechanism. The composition at *z* = 1.73, *n* < 0.5, was classified as “small Fick’s diffusion” [44]. It was also seen that with an increase in the *z* value to 1.73, the diffusion coefficient decreased by about 25%, correspondingly. This feature emphasized that the diffusion of the solvent into the hydrogel matrix was complicated by increasing the crosslinking density of the hydrogel. Also, based on the values of the swelling rate constant according to the pseudo-second-order model (Figure 7b), it can be noted that at *z* > 1 there was a significant deceleration of water absorption at the initial stage of the process. This also indirectly confirmed the increased efficiency of polyelectrolyte binding.

In the case of tests in the *PBS* and *SGF* solutions, the sorption parameters changed coordinately. Table 2 shows that the diffusion coefficient *n* for all *z* values was less than 0.5. It is noteworthy that the increase in the *z* value was largely not reflected in the values of the diffusion coefficient and had a low level. This effect shows that the diffusion of the buffer solution into the hydrogel matrix was difficult for the reasons described above. It is also evident that with an increase in the *z* value, within the pseudo-second-order model, the sorption rate constant value *k*_2_ increased (Figure 7d,f), indicating the acceleration of the swelling process at the initial stage of the test.

## 3. Conclusions

In this work, the process of the self-assembly of (bio)PEC hydrogels between chitosan and gelatin in a wide range of ratios of oppositely charged ionized groups (*z*) was studied. It was shown that sufficient conditions for the gelation of primary coacervate structures into hydrogel material are 25 °C in the ratio range of 0.58 ≤ *z* ≤ 1.73.

The analysis of the chemical structure of films obtained by the melt casting of the (bio)PEC hydrogels using FTIR spectroscopy showed shifts in the absorption bands of amino groups in chitosan and carboxyl groups in gelatin. These shifts may have been due to electrostatic interactions between the polymers under the selected formulation conditions. In addition, at *z* ≥ 1, a 12% increase in the intensity of the broad absorption band in the wave number range 3600–3000 cm^−1^ was observed, indicating an increase in intra- and intermolecular hydrogen interactions.

The investigation of the elastic properties of the condensed hydrogels by dynamic mechanical analysis revealed a three-fold increase in the elastic properties and crosslinking density, as well as a decrease in the distance between the crosslinking points by 43%, with an increase in the *z* values to 1. It is noteworthy that a significant decrease in the rate of increase in the elastic component was observed when the proportion of gelatin in the system was increased at *z* ≥ 1, indicating that the limit of polyelectrolyte binding between the polymers had been reached, leading to the formation of a more isotropic system.

The sorption analysis of the (bio)PEC lyophilizates in distilled water showed a 45% decrease in the equilibrium degree of swelling and a decrease in the sorption rate constant by more than an order of magnitude when increasing the proportion of gelatin in the system to *z* = 1.73. This further confirmed the correlation between the *z* values and crosslinking density in the hydrogel compositions. It is also significant that the analysis in the *PBS* and *SGF* solutions revealed a two- to three-fold decrease in the equilibrium degree of swelling for each composition compared to the distilled water. Equally remarkably, an opposite trend was observed with a 50% increase in the equilibrium degree of swelling and a more than four-fold increase in the sorption rate constant as the *z* values increased, up to 1.73, when the samples were swollen in the *PBS* solution. This effect may have been related to the amount of charged carbonyls on the hydrogel surface, which increased with the increasing *z* and decreased under the action of low-molecular-weight cations in the solution. For samples in the *SGF* solution with *z* ≥ 0.89, despite the acidic pH of the medium, a relatively low loss of hydrogel mass was observed, which also proved the efficiency of the formation of (bio)PEC structures at these polymer ratios.

In vitro studies of the enzymatic resorption of hydrogel samples in a solution of *PBS* with lysozyme at 37 °C showed that the character of the mass loss of the sample depended on the *z* values. At *z* = 0.89 and 1.15, the stability of the sample increased; the samples were slowly resorbed for 2 days and did not lose weight during the 300 min of the experiment. This proves the fact that the Chit–Gel (bio)PEC hydrogels at *z* ≈ 1 had the greatest resistance to the aggressive conditions of the liquid medium. The prospects for further research lie in the development of (bio)PEC structures that are more resistant to aggressive environments and human body temperature.

## 4. Materials and Methods

### 4.1. Materials

We used gelatin type B, produced by Sigma-Aldrich Corporation (St. Louis, MO, USA) (CAS 9000-70-8) with an average viscous molecular weight of *M_v_* = 63.1 ± 8.7 kDa and an isoelectric point of *pI* = 4.7 ± 0.3, which were determined by viscometric and turbidimetric methods in previous work [27]. The chitosan was produced by CJSC “BioProgress” (Moscow, Russia), with *M_v_* = 206 ± 1.5 kDa; a protonation constant *pK*_0_ = 6.35; and a degree of deacetylation of DD = 83.1% [45]. The glacial acetic acid and sodium hydroxide were produced by JSC “LenReactiv” (Saint Petersburg, Russia), and we also used distilled water (in accordance with the standard of GOST R 58144–2018 [46]).

### 4.2. Preparation of Self-Assembled Chitosan–Gelatin (Bio)PEC Hydrogels

To prepare the (bio)PEC hydrogels, individual solutions of chitosan (Chit) with a concentration of *C* = 1 wt.% in 0.2 M CH_3_COOH and gelatin (Gel) with *C* = 4 wt.% in distilled water were prepared, which were mixed in ratios of chitosan–gelatin (materials have been coded as Chit–Gel) of 1:10, 1:15, 1:20, 1:25, and 1:30. The preparation of the solutions of individual biopolymers was carried out by dissolving the powders for 24 h at a temperature of 35 ± 0.2 °C with constant stirring with a magnetic stirrer to complete the dissolution. The required temperature of the solution was maintained using an ETS-D6 probe thermostat IKA-Werke (Baden-Württemberg, Germany) coupled with a magnetic stirrer. Further, the obtained polymer solutions were mixed by weight for 1 h at a temperature of 35 ± 0.2 °C with constant stirring in the following ratios: 1:1; 1:5; 1:10; 1:15; 1:20; 1:25; and 1:30. The resulting mixtures were titrated with a solution of 2 M NaOH to pH = 6. As a result, dispersions of (bio)PEC particles were obtained, which were thermostated for 1 h at a temperature of 35 ± 0.2 °C until the appearance of sediment and then settled for 24 h at room temperature to form a hydrogel. A hydrogel based on an individual Gel solution was also obtained according to the above methodology.

The composition of the (bio)PEC hydrogels were expressed as the molar ratio of the ionized functional side groups *z*:

(1) $$z=\frac{\left[COO^{-}\right]}{\left[NH_{3}^{+}\right]},$$

where [*COO*^−^] (for Gel) and [*NH*_3_^+^] (for Chit) is the molar concentration of charged units.

The values of *z* were calculated taking into consideration the *DD* of chitosan and the amino acid composition of gelatin. Based on this, for the chitosan macromolecule, 83.1% of all units had a positive charge on the amino groups of *D*-glucosamine residues. For the gelatin, it was approximately 7.2 and 4.8% of the negatively charged carboxyl groups in the residues of glutamic and aspartic acids, correspondingly [20]. For the studied ratios of the chitosan–gelatin (bio)PEC, the calculated *z* values were 0.06, 0.29, 0.58, 0.89, 1.15, 1.44, and 1.73, correspondingly. The mass yield of the (bio)PEC structures was determined as the ratio of the mass of the initial polymer powders to the mass of the precipitated lyophilized hydrogel sample.

### 4.3. Scanning Electron Microscopy of Hydrogels

To study the morphology of the hydrogels, the samples were lyophilized at −35 °C and a vacuum pressure of 1.65 mPa for 72 h in a FreezeDry TRIAD lyophilizer (LABCONCO, Kansas, MO, USA). The surface morphology of the obtained lyophilizates was characterized using a scanning electron microscope MIRA-3, Tescan (Brno, Czech Republic), at a voltage of 2 kV. The obtained electron micrographs showed the highly porous structure of the hydrogels because of which they were analyzed statistically with the determination of the diameter using Image-J v. 2.1.0 software, National Institutes of Health (Bethesda, MD, USA).

### 4.4. Attenuated Total Reflectance Fourier-Transformed Infrared Spectroscopy of Hydrogels

The chemical composition of the (bio)PEC hydrogels analyzed using an ATR-FTIR spectrometer, Tensor 37, produced by Bruker (Bremen, Germany) and equipped with an attenuated total reflection module (ATR), MIRacle, Pike technologies (Madison, WI, USA). The data were recorded in the range of 4000–600 cm^−1^ with a resolution of 2 cm^−1^, and averaging over 32 spectra was conducted using OPUS software v. 7.8.

### 4.5. Thermogravimetric Analysis of Hydrogels

The hydrogel samples were subjected to thermogravimetric analysis (TGA) to assess their moisture content using a thermobalance machine, TG 209 F1 Libra Netzsch (Selb, Germany), in an inert atmosphere (super-dry nitrogen gas) with a constant flow rate of 250 mL/min. Sample preparation included obtaining samples of a similar geometry with a mass of 10 ± 0.2 mg before the measurement, which were conditioned in a desiccator at a relative humidity of 80% for 72 h. Weight loss was estimated at a heating rate of 10 °C/min in the temperature range from 25 to 200 °C. The measuring crucible for this test was made of Al_2_O_3_ which did not change its weight in the investigated temperature range.

### 4.6. Dynamic Mechanical Analysis of Hydrogels

The viscoelastic properties and structure of the hydrogels were analyzed using a Physica MCR 502 rheometer, Anton Paar (Ankerstraße, Austria), equipped with a parallel plate measuring system, PP25 with an upper plate diameter of 25.003 mm. After the preparation of the hydrogels, according to the procedure described above, the test samples were placed in Petri dishes to avoid additional deformations and stresses before the test. Sample preparation included conditioning in a desiccator at a relative humidity of 80% for 72 h. The experimental data of the storage modulus (*G*′) and loss modulus (*G*″) were recorded with a constant value of angular frequency of 10 Hz in wide range of shear strain, *γ* = 0.001–500%, at a temperature of 25.0 ± 0.2 °C. The hydrogels were thermostated during the tests using a C-PTD200 module equipped with Peltier elements.

Based on the data of the dynamic mechanical analysis (DMA), the distances between the crosslinking points, ζ (network size), and the crosslinking density of *n_e_* were determined according to Equations (2) and (3) [47]:

(2) $$\xi ={\left(\frac{G_{e}N_{A}}{RT}\right)}^{-1/3},$$

(3) $$n_{e}=\frac{G_{e}^{\prime }}{RT},$$

where *G*′*_e_* is the average plateau value of *G*′; *N_A_* = 6.022 × 10^23^ is the Avogadro number; *R* = 8.314 J/Kmol is the universal gas constant; and *T* is the absolute temperature.

### 4.7. Sorption Kinetics Analysis of Hydrogels

To study the liquid sorption process of the obtained hydrogels, samples with the same geometry were prepared, which were then lyophilized according to the method described in Section 2.2.

The swelling process was studied by a gravimetric method in distilled water and a phosphate-buffer saline (*PBS*) solution. The *PBS* solution (pH = 7.4) contained 137 mM NaCl, 2.7 mM KCl, 10.1 mM dibasic sodium phosphate, and 1.8 mM monobasic potassium phosphate [48]. Simulated gastric fluid (*SGF*) buffer was prepared by dissolving 2.0 g of sodium chloride in 7 mL of 37% aqueous hydrochloric acid solution, followed by the dilution of the mixture to 1000 mL [49]. In vitro enzymatic resorption was performed in a *PBS* buffer containing 10 mg/L of lysozyme to simulate the environment of the human body at a temperature of 37 °C. Measurements were carried out on three samples for each hydrogel composition to determine the standard deviation of the obtained results. The degree of swelling, *Q*, was determined by Equation (4) and characterized the amount of liquid absorbed by 1 g of lyophilized hydrogel [50]:

(4) $$Q=\frac{m_{s}-m_{0}}{m_{0}},$$

where *m_s_* is the mass of the swollen sample, g; and *m*_0_ is the mass of the dried sample, g.

To determine the degree of swelling, the tested samples of the hydrogel material were placed in a container with liquid at room temperature, from which they were subsequently extracted. After the extraction of the swollen hydrogel sample, before weighing on analytical scales, the moisture on the surface of the material was removed by soaking.

The Korsmeyer–Peppas model (5) was used to describe the diffusion of liquid into the hydrogel matrix [51]:

(5) $$\frac{Q_{t}}{Q_{\infty }}=kt^{n},$$

where *Q_t_* and *Q_∞_* are the degree of swelling at a time *t* and the equilibrium degree of swelling, respectively; *n* is Fick’s constant, which determines the type of diffusion; and *k* is a constant associated with the structure of the polymer network.

Also, the pseudo-second-order kinetic model was used:

(6) $$Q_{t}=\frac{t}{\frac{1}{k_{2}\times Q_{\infty }^{2}}+\frac{t}{Q_{\infty }}},\;$$

where *k*_2_ is the sorption rate constant of the pseudo-second order model, g·(mg·min)^−1^; *t* is time, min; *Q_t_* is the sorption capacity at a time *t*, g; and *Q_∞_* is the equilibrium degree of the swelling capacity, g.

After the sorption analysis, the kinetic parameters of moisture absorption were calculated according to Equations (5) and (6). The calculation of the exponent *n* and the constant *k* was performed by plotting the data after the logarithm of the *Q_t_* values, followed by estimation using functions.

## Figures and Tables

**Table 1:** Structural parameters of Gel and Chit–Gel (bio)PEC hydrogels at different *z* values.

Chit–Gel	*z*	Moisture Content, %	*G_e_*′, Pa	LVE Limit, %	ζ, nm	*n_e_*, mol/m^3^
0:1	-	-	480 ± 21	78.2	20.7	0.186
1:10	0.58	93	778 ± 16	4.2	17.6	0.303
1:15	0.89	90	1457 ± 10	1.3	14.3	0.565
1:20	1.15	89	2249 ± 17	1.9	12.4	0.872
1:25	1.44	91	2269 ± 12	1.5	12.4	0.880
1:30	1.73	91	2318 ± 16	1.9	12.3	0.900

*z*—molar ratio of ionized functional side groups; *G_e_*′*—*plateau value of storage modulus; ζ—distance between the crosslinking points; *n_e_*—crosslinking density.

**Table 2:** Sorption characteristics of Chit–Gel bio(PEC) hydrogel lyophilizates in distilled water, *PBS*, and *SGF* solutions.

Chit–Gel	*z*	*Q_max_*, g/g	*n*	*k*	*k*_2,_ g·(mg·min)^−1^	*R* ^2^ *_k_*_2_
Distilled water						
1:15	0.89	31	0.59	0.037	0.752	0.99
1:20	1.15	27	0.61	0.024	0.162	0.99
1:25	1.44	20	0.57	0.032	0.078	0.99
1:30	1.73	17	0.44	0.062	0.049	0.99
PBS solution						
1:15	0.89	8	0.29	0.11	0.003	0.99
1:20	1.15	10	0.29	0.11	0.006	0.99
1:25	1.44	11	0.28	0.12	0.007	0.99
1:30	1.73	12	0.25	0.16	0.014	0.99
SGF solution						
1:15	0.89	7	0.26	0.17	0.003	0.99
1:20	1.15	8	0.30	0.15	0.006	0.99
1:25	1.44	8	0.20	0.28	0.009	0.99
1:30	1.73	9	0.18	0.29	0.009	0.99

*z*—molar ratio of ionized functional side groups; *Q_max_*—sorption capacity; *n*—Fick’s constant; *k*—constant associated with structure of polymer network; *k*_2_—sorption rate constant.

## Data Availability

The data presented in this study are available on request from the corresponding author.
